# Identification of Candidate Genes Involved in Curd Riceyness in Cauliflower

**DOI:** 10.3390/ijms21061999

**Published:** 2020-03-15

**Authors:** Zhenqing Zhao, Xiaoguang Sheng, Huifang Yu, Jiansheng Wang, Yusen Shen, Honghui Gu

**Affiliations:** Institute of Vegetables, Zhejiang Academy of Agricultural Sciences, Hangzhou 310021, China; zhaozq@zaas.ac.cn (Z.Z.); xguang@zaas.ac.cn (X.S.); hfyu@zaas.ac.cn (H.Y.); wangjs@zaas.ac.cn (J.W.); yusen612@163.com (Y.S.)

**Keywords:** cauliflower, curd development, riceyness, genetic mapping, flowering regulation

## Abstract

“Riceyness” refers to the precocious development of flower bud initials over the curd surface of cauliflower, and it is regarded as undesirable for the market. The present study aimed to identify the candidate loci and genes responsible for the morphological difference in riceyness between a pair of cauliflower sister lines. Genetic analysis revealed that riceyness is controlled by a single dominant locus. An F_2_ population derived from the cross between these sister lines was used to construct “riceyness” and “non-riceyness” bulks, and then it was subjected to BSA-seq. On the basis of the results of Δ(SNP-index) analysis, a 4.0 Mb candidate region including 22 putative SNPs was mapped on chromosome C04. Combining the RNA-seq, gene function annotation, and target sequence analysis among two parents and other breeding lines, an orthologous gene of the *Arabidopsis* gene *SOC1*, *Bo4g024850* was presumed as the candidate gene, and an upstream SNP likely resulted in riceyness phenotype via influencing the expression levels of *Bo4g024850*. These results are helpful to understand the genetic mechanism regulating riceyness, and to facilitate the molecular improvement on cauliflower curds.

## 1. Introduction

Curd of cauliflower (*Brassica oleracea* var. *botrytis*) is constituted by a mass of spirally arranged floral branches with whorls of inflorescence and floral meristems, the whole held tightly together and forms a hypertrophied corymb [1,2]. Typical cauliflower bears a semi-spherical curd with fairly smooth and rounded surface. However, during curd growth and development, initially developmental buds often appear on the curd surface, which is known as “riceyness” (Figure S1). It is one of the most serious risks in cauliflower production, because riceyness influences on both quality and yield of curd.

The initiation and development of curd occurs at the early reproductive stage of cauliflower, which is regarded as a floral reversion phenomenon [3]. At the curd development stage, the ability to produce mature floral structures is temporarily inhibited [4,5], while the iterative process of proliferations of apical meristems of branches induces a rapid increase in curd size [6] Some pedicels start bolting accompanied with the differentiation of floral meristems only when the curd develops to the post-maturity stage, and then the plant begins to bloom [3,6]. Therefore, the essence of riceyness is that the partial or total ability to differentiate floral meristems is restored in advance of the curd development stage, which is closely related to the regulatory network of plant flowering, especially the determinacy mechanism of floral meristems.

Plant flowering regulation is controlled by an intricate network of genetic regulators and their interaction with environmental factors [7]. Similarly, riceyness is thought to be influenced, to some extent, by the environmental conditions. However, it is also worth noting that different varieties have shown various levels of resistance to riceyness, suggesting that this undesirable trait is genetically controlled by polygenes or major genes whose effects were modified by environmental factors [8]. Among the flowering regulatory genes, floral meristem identity genes A*PETALA1* (*AP1*) and *CAULIFLOWER* (*CAL*) were intensively studied in cauliflower, as the *ap1 cal* double mutants of *Arabidopsis* form floral meristems similar to cauliflower curds [5]. In *Arabidopsis*, *AP1* and *CAL* belong to the MADS-box regulatory gene family and have the function of promoting the flowering process [9]. Because of the strong similarity of amino acid sequences and a highly redundant biological function between *AP1* and *CAL*, curd-like organs can only be formed when both of these two genes are mutated in *Arabidopsis* [5]. In cauliflower, a terminated mutation in the fifth exon of *CAL* results in the functional deficiency of its encoding product, whereas *AP1* has normal function [10]. Subsequent studies have also shown that mutant *CAL* allele is present in different cauliflower varieties from different regions, suggesting that *CAL* is responsible for the cauliflower phenotype in *Brassica oleracea* species [11]. Interestingly, there is also evidence that there are two copies of *CAL* gene, including one mutant *CAL-T* and one wild *CAL-G,* coexisting in the riceyness cauliflower and broccoli, indicating that riceyness could be related to the sequence or functional variation of the *CAL* gene in cauliflower [12]. However, when the full-length exogenous *CAL* gene is introduced into cauliflower, the transgenic lines give rise to inflorescence composed of green flower buds and lose the ability to form curds [13], which are morphologically different from riceyness. So far, the molecular mechanisms of riceyness are not clear. Genes and loci associated with riceyness are not well revealed. In cauliflower breeding, the traditional methods based on phenotypic selection are still the main way to improve tolerance for riceyness of the cultivar [14,15 and 16].

In this paper, we subject bulked segregant analysis using the sequencing (BSA-seq) method and RNA-seq analysis in order to perform the gene mapping and candidate gene identification of riceyness using a pair of sister lines showing morphological differences in riceyness tolerance.

## 2. Results

### 2.1. Phenotypic Variation and Genetic Analysis

**Figure 1:**
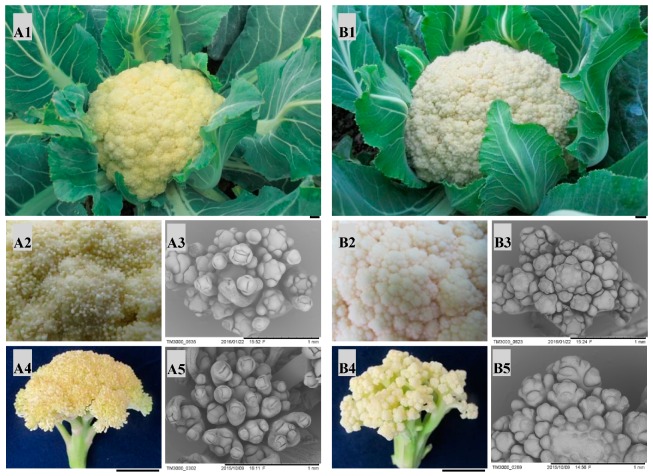
Phenotypic differences between” QNF11-3” (**A**) and ”QNF11-16” (**B**). A1 and B1, curds appearance at economical maturity stage; A2 and B2, enlarged view of the meristems on the curd surface at economical maturity stage; A3 and B3, electron microscope images of the surface meristems at economical maturity stage; A4 and B4, appearance of surface meristems when the plants start bolting; A5 and B5, electron microscope images of the surface meristems when the curds start bolting. The black bars in the lower right in A1, B1, A4, and B4 indicate 1 cm, and those in A3, B3, A5, and B5 indicate 1 mm.

Two parents (QNF11-3 and QNF11-16) showed high similarity in botanical morphology except curd morphology. There were numerous canary yellow ricey tissues covering on the curd surface of QNF11-3, whereas the curd of QNF11-16 showed a smooth surface (Figure 1). Scanning electron microscope observation revealed the ricey tissues were floral meristems including many first-round floral organs, indicating that QNF11-3 gains the ability to differentiate floral organs at the curd stage without the elongation of the pedicels. These results suggested that the riceyness phenotype could be related to the regulation of plant flowering.

Phenotypic variations were also visible among segregation populations. Table 1 shows the phenotypic segregation data of all generations. Two reciprocal crossing F_1_ and one BC_1_ generated from QNF11-3×F_1_ displayed non-segregation with uniform riceyness phenotype. The riceyness and non-riceyness segregation of F_2_ population and another BC_1_ population (QNF11-16×F_1_) accorded with the expectable 3:1 (χ^2^ = 0.18, *p* > 0.05) and 1:1 (χ^2^ = 0.75, *p* > 0.05), respectively, indicating a monogenic inheritance. Riceyness is dominant to non-riceyness.

### 2.2. Genetic Mapping of Riceyness

**Figure 2:**
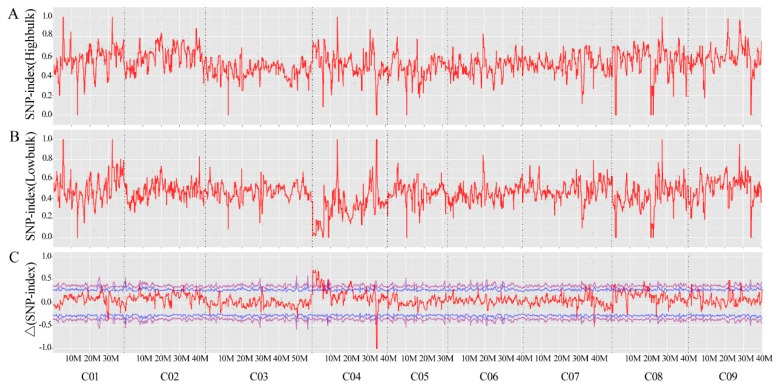
SNP-index graphs of bulk-R (**A**) and bulk-N (**B**), and Δ(SNP-index) graph (**C**) based on BSA-seq analysis. The X-axis represents the position of genome and the Y-axis represents the SNP-index for A and B, and Δ(SNP-index) for C. A candidate region was identified in chromosome C04 (3 to 7 Mb interval) with the peak position at 4.01 Mb.

A total of 2,24,791,210 and 1,89,137,118 clean reads was generated from QNF11-3 (53.05X mean depth coverage) and QNF11-16 (44.28X mean depth coverage), respectively, while 206,281,340 and 205,882,398 clean reads were generated from bulk-R (48.77X mean depth coverage) and bulk-N (48.94X mean depth coverage), respectively. After the reads were compared with the reference genome sequence, 3,897,753 and 3,887,134 SNPs were identified in QNF11-3 and QNF11-16, respectively, while 3,894,335 and 3,893,700 SNPs were identified in bulk-R and bulk-N, respectively. According to the SNP-index and Δ(SNP-index) of these SNPs, only one region that was on chromosome C04 from 3 to 7 Mb exhibited a significant difference between bulk-R and bulk-N (Figure 2). The peak position was at 4.01 Mb on chromosome C04, and the corresponding Δ(SNP-index) reached 0.78 (Figure 2). This finding was consistent with the assumption that the riceyness and non-riceyness difference between QNF11-3 and QNF11-16 was controlled by a single locus. SNP ratio of each SNP among this region for each parent and each bulk was calculated in order to speculate the putative SNPs associated with riceyness. In total, 22 SNPs accorded with the expected ratio of a monogenic inheritance model; of these, three SNPs located at gene coding sequences, whereas the other 19 mutations located at upstream, downstream, or intergenic regions of function genes (Table S1). These SNPs were associated with a total of 29 genes (Table S1).

### 2.3. Transcriptome Differences between Ricey and Non-Ricey Parents

To acquire insights into the transcriptomic differences between the ricey and non-ricey parental lines, RNA-seq analysis was performed between the curd tissues of QNF11-3 and QNF11-16. About 298 million clean reads were obtained from six libraries and were used for quantitative gene expression analysis; 90.73% to 91.04% of the clean reads could be mapped to predict gene regions, including 88.48% to 88.88% unique mapped reads, and 2.12% to 2.43% multiple mapped reads. The detailed data of sequencing is shown in Table 2.

**Figure 3:**
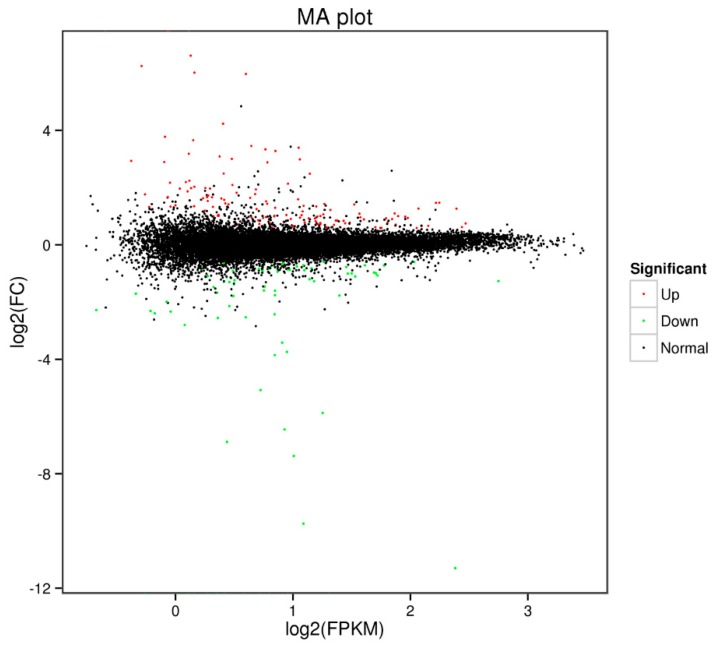
Scatter diagram of the differentially expressed genes. The green dots represent downregulated genes, the red dots represent upregulated genes, and the black dots represent non-differentially expressed genes.

Differential expression analysis revealed a total of 213 DEGs. In ricey parent QNF11-3, 128 genes were upregulated expressed and 85 genes were downregulated expressed as compared with the non-ricey parent QNF11-16 (Figure 3; Table S2).

### 2.4. Identification of Candidate Genes

Out of the 29 genes identified by genetic mapping, only *Bo4g024850* encoding an MADS-box protein *SOC1* (suppressor of overexpression of *CO1*), showed significantly different expression levels between two parents. The corresponding SNP (coded as SNP-5) located at the upstream of *Bo4g024850*, and its physical position (4,029,234 bp on C04) was very close to Δ(SNP-index) peaked for the riceyness trait (Figure 2). It has also been reported that overexpression of *SOC1* (also known as *AGL20*) not only suppressed the late flowering of plants that had functional *FRI* and *FLC* alleles but also promoted flowering and inflorescence meristem identity in *A. thaliana* [17,18]. Thus, *Bo4g024850* was presumed to be the most likely candidate gene responsible for the riceyness phenotype, and the morphological difference of curd between two parents possibly resulted from the upstream SNP-5, which could have functions of regulating the expression levels of *Bo4g024850*.

**Figure 4:**
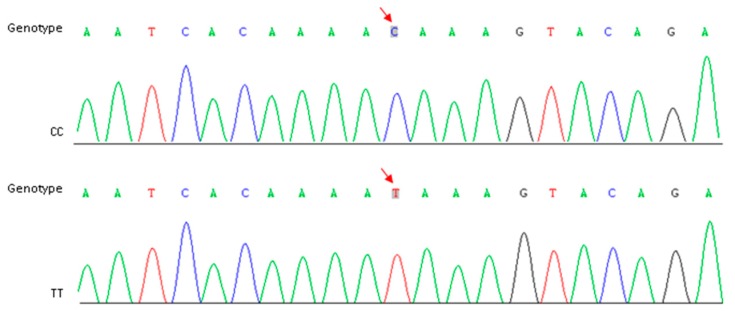
Identification of CC and TT genotype in parents and other breeding lines. Red arrows indicate the position of SNP-5.

**Figure 5:**
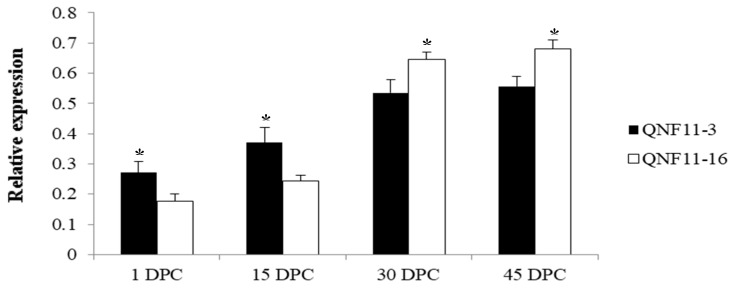
Expression levels of *Bo4g024850* between QNF11-3 and QNF11-16 at different curd developmental stages. The X-axis represents the sampling period (days post curding, DPC). Error bars represent standard errors derived from three replications and asterisks represent significant differences (* *p* < 0.05).

In order to reconfirm our assumption, a specific primer pair (Table 3) was designed to amplify the target fragment including SNP-5 in two parents and the other 10 cauliflower inbred lines. The purified PCR products were sequenced using the Sanger method. Sequencing results showed that this C-to-T transversion existed widely within the plant materials we detected, and the genotypes were perfectly matched to the riceyness phenotype (Figure 4 and Table 4). Similarly, another SNP (SNP-3) located at gene coding sequences of a functionally uncharacterized gene (*Bo4g023880*) was also detected among these materials, but it did not show a clear correlation between genotype and phenotype (Table 4). Furthermore, we further validated the expression levels of *Bo4g024850* using qRT-PCR in curd tissues of different curd developmental stages (1, 15, 30, and 45 days post curding, respectively) of QNF11-3 and QNF11-16. The expression levels of *Bo4g024850* in QNF11-3 and QNF11-16 were increasing continuously along with the curd development (Figure 5). For samples from one day and 15 days post curding, the expression level of *Bo4g024850* in QNF11-3 was higher than that in QNF11-16, which were consistent with results of RNA-seq analysis. However, opposite results were gained during the other two sampling periods (Figure 5).

## 3. Discussion

Riceyness is one of the major quality defects of cauliflower curds. Biologists and breeders have been trying to find effective ways to genetically improve this undesirable trait. Herein, we used a pair of ricey and non-ricey sister lines to identify the genomic regions and candidate genes responsible for the riceyness trait. The results gained should be helpful to understand the genetic mechanism regulating riceyness, and to facilitate the molecular improvement on cauliflower curds.

BSA-seq analysis revealed a total of 22 SNPs related to riceyness, which gave a clear indication for candidate genes identification. Similarly, only 213 DEGs between two parents were identified via transcriptome sequencing. This number is far less than that gained in several recent transcriptome studies on *Brassica oleracea* species [19,20], but similar to the 306 DEGs uncovered between a pair of near-isogenic lines of cotton [21]. Such a limited number of candidate SNPs and DEGs were attributed to the highly uniform genetic background between two parents, which could have filtered out a lot of false positives, and therefore significantly enhanced the efficiency of BSA-seq and RNA-seq analysis.

By using BSA-seq analysis, 19 SNPs located at upstream, downstream, or intergenic regions of function genes and three SNPs located at gene coding sequences were identified. The three SNPs located at gene coding sequences resulted in missense variants in *Bo4g023880*, *Bo4g026630*, and *Bo4g026830*, respectively. We could not exclude these three genes as candidates, although there were no obvious expression level differences of them between the two parents. However, gene annotation indicated that *Bo4g026630* and *Bo4g026830* encoded a myosin-11 protein and inorganic phosphate transporter 1–4-like proteins, respectively, which seemed hard to associate with riceyness. In contrast, *Bo4g023880* was a functionally uncharacterized gene, and then was regarded as a candidate gene together with *Bo4g024850*. Nevertheless, the SNP existing in *Bo4g023880* (SNP-3) did not show a correlation with riceyness phenotype among the other breeding lines (Table 4). Jointly, as the orthologous gene of *SOC1* in *Brassica oleracea*, *Bo4g024850* was presumed as the candidate gene responsible for the riceyness. In any case, this assumption needs to be verified via further fine mapping and transgenic analysis.

As one of the floral integrator genes, *SOC1* plays an important role during the process of phase transition in plants from vegetative to the reproductive stage [22]. The overexpression of *SOC1* resulted in early flowering, whereas *soc1* mutant plants showed later flowering than the wild type *Arabidopsis thaliana* [18]. A series of studies have also revealed that *SOC1* is a multifunctional gene which not only triggers the floral transition but also regulates floral patterning and floral meristem determinancy [23,24,25]. *SOC1* is known to induce floral meristem identity gene *LFY* expression at the shoot apex, thus promoting the establishment and maintenance of floral identity in emerging floral meristems [22,26]. However, *SOC1* does not maintain high expression throughout the whole flowering process. In contrast to the maintained strong *SOC1* signals within the inflorescence meristem, its expression in developing young floral meristems and stage one and two flowers is turned off [27,28]. It is suggested that *SOC1* is repressed by multiple factors including *AP1* [23] and *SEP3* [29], when the floral meristem identity has been established and maintained. In the present study, it is interesting to note that the expression signal of *Bo4g024850* in QNF11-3 was stronger than that in QNF11-16 during early curd development (1DPC and 15 DPC), but weaker during late curd development (30 DPC and 40 DPC). This trend is consistent with previous research results, because there has already been a mass of floral meristems and flower buds on the curd surface in QNF11-3 but only inflorescence meristems in QNF11-16 (Figure 2), indicating that floral identity in QNF11-16 has not been established. Furthermore, the candidate SNP-5 located at about 4000 bp upstream of *Bo4g024850* transcription start site. The mechanism of SNP-5 long distance regulating *Bo4g024850* expression in cauliflower curd riceyness is also an interesting issue to be explored.

Curd riceyness is not only an economical factor in cauliflower production, but also a special form of plant flowering regulation. The candidate loci and genes uncovered in the present study provide powerful tools for cauliflower molecular breeding and enrich people′s understanding of the regulatory network controlling plant flowering.

## 4. Materials and Methods

### 4.1. Plant Materials and Phenotype Determination

”Qingnong65” is a cauliflower commercial cultivar widely cultivated in China. When the temperature during the curd development period is below 12 °C, its curd shows different degrees of riceyness. QNF11-3 and QNF11-16 are a pair of sister lines generated from an individual of an F_7_ inbred line originated from “Qingnong65” (Figure S2). This pair of sister lines show high uniformity in agronomic traits and genetic background but significantly morphological differences in riceyness tolerance, especially at low temperatures. In a multiple years and sites phenotype survey, QNF11-3 showed riceyness curd, whereas QNF11-16 showed smooth curd (Figure 1). F_1_ plants were generated by reciprocal cross between QNF11-3 (female/male) and QNF11-16 (male/female). A single F_1_ individual from the cross of QNF11-16 (female) and QNF11-3 (male) was, then, used to produce F_2_ and BC_1_ populations. All the entries were seeded in growing matrix on 10 October, 2018. Then, 25-day seedlings were planted into a greenhouse in Haining County (HN, 30°320 N, 120°410 E) with 50 cm row spacing and 65 cm line spacing. When the curd diameter reached 10 cm, the phenotype of each individual was identified by visual observation and classified as “riceyness” and “non-riceyness”.

### 4.2. Genomic DNA Isolation and Bulking

On the basis of the phenotype determination, 38 individuals showing remarkable riceyness and 50 individuals with unambiguous non-riceyness curd from the F_2_ population were selected to construct the extreme phenotype bulks for BSA-seq. Genomic DNA of these F_2_ individuals and parents was isolated from fresh leaves using a DNA Secure Plant Kit (Tiangen, Beijing, China). DNA concentration and quality were measured by an ND-1000 spectrophotometer (NanoDrop, Wilmington, DE, USA) and electrophoresis on 1.0% agarose gel with a standard lambda DNA. Riceyness bulk (bulk-R) and non-riceyness bulk (bulk-N) were then constructed by pooling equal quantity of DNA from 38 riceyness individuals and 50 non-riceyness individuals.

### 4.3. Genome Sequencing and Analysis

The BSA-seq was performed on the Illumina HiSeq X10 platform (Oebiotech, China) following the detailed procedure as described by Wang et al. (2018). After filtering the low-quality raw data using NGSQC toolkit software, the clean reads of bulk-R and bulk-N were aligned to the *Brassica oleracea* reference genome sequence (https://www.ncbi.nlm.nih.gov/ assembly/GCF_000695525) using BWA software. SAM tools were used to perform single-nucleotide polymorphism (SNP) calling. The SNP-index and Δ(SNP-index) were calculated to identify candidate regions associated with the riceyness trait [30].

The SNP ratio (the ratio of reads number different from the reference base to the total reads number at this locus) of each SNP detected within the candidate region were calculated for bulk-R, bulk-N, and two parents, respectively. On the basis of the genetic analysis of riceyness in F_2_ and BC_1_ population, a monogenic inheritance model was used to identify the putative SNPs associated with riceyness. The criterion is that SNP ratio should be higher than 0.9 for QNF11-3, higher than 0.5 for bulk-R, lower than 0.1 for QNF11-16, and lower than 0.2 for bulk-N [31].

### 4.4. RNA-Seq Analysis

The curd tissue of parental line QNF11-3 and QNF11-16 was respectively collected at the early curding stage (1 day post curding), to isolate total RNA using a Plant RNA Mini Kit (Tiangen, Inc., China). Three biological replicates were performed for each sample (T01, T02, and T03 for QNF11-3; T04, T05, and T06 for QNF11-16). A total amount of 1 μg purified RNA per sample was subjected to construct the cDNA libraries using a NEBNext UltraTM RNA Library Prep Kit for Illumina (NEB, Inc., USA). The library quality was assessed on the Agilent Bioanalyzer 2100 system (Agilent Technologies, Inc., Santa Clara, CA, USA).

The six library preparations were subsequently sequenced on an Illumina HiSeq2500 platform and paired-end reads were generated. Data analysis was performed following the procedures described by Jian et al. (2019) [32]. Generally, then, the high-quality reads were mapped to the *Brassica oleracea* reference genome sequence using HISAT2; only reads with a perfect match or one mismatch were further analyzed and annotated based on the reference genome. Gene expression levels were estimated by fragments per kilobase of transcript per million fragments mapped (FPKM). Differential expression analysis between two parental lines was performed using the DEseq. The resulting *p* values were adjusted using the Benjamini and Hochberg’s approach for controlling the false discovery rate. Genes with an adjusted *p*-value < 0.05 found by DEseq were assigned as differentially expressed [33]. Significant differentially expressed genes (DEGs) were determined based on a threshold of 1.2-fold expression change.

For qRT-PCR, specific primers (5′-acaaactgagcagcccaagca-3′ and 5′-ctcgtcgtcgcctcttccac-3′) were designed based on the cDNA sequence of *SOC1*. qRT-PCR was conducted in an ABI StepOne-Plus machine using SYBR® Premix Ex Taq™ (TaKaRa, Shiga, Japan). The detailed reaction system and PCR procedure was performed as that described by our previous paper [34]. The 2^−ΔCt^ value was used to measure the relative expression levels for putative SNPs validation.

In order to further validate if the putative SNPs were associated with riceyness phenomenon, putative SNPs were detected in two parents and other 10 cauliflower inbred lines (saved in Zhejiang academy of agricultural sciences) using the Sanger sequencing method. The 250 bp sequences flanking each candidate SNP on either side were used to design the PCR primers. The purified PCR products were sequenced following the chain termination protocol at Sangon Biotech (Shanghai) Co., Ltd.

## Figures and Tables

**Table 1:** Phenotypic segregation and genetic analysis of the riceyness trait among all populations constructed in the present study.

Accessions	Total	Riceyness	Non-Riceyness	Presumptive Segregation Ratio	χ^2^
P1 (QNF11-3)	24	24	0		
P2 (QNF11-16)	24	0	24		
F_1_-1 (P1×P2)	24	24	0		
F_1_-2 (P2×P1)	24	24	0		
BC_1_-1 (P1×F_1_-2)	128	128	0		
BC_1_-2 (P2×F_1_-2)	162	87	75	1:1	0.75
F_2_ (F_1_-2selfing)	188	138	50	3:1	0.18

χ^2^ 0.05,1 = 3.841.

**Table 2:** Summary of the RNA-seq data for the six samples.

Sample ID	Clean Reads	GC Content	≥Q30 Percent	Mapped Reads	Unique Mapped Reads	Multiple Mapped Reads
QNF11-3a	54,571,970	47.97%	93.25%	49,513,194 (90.73%)	48,304,718 (88.52%)	1,208,476 (2.21%)
QNF11-3b	45,719,248	48.03%	94.01%	41,623,020 (91.04%)	40,510,608 (88.61%)	1,112,412 (2.43%)
QNF11-3c	54,239,870	48.00%	93.50%	49,331,459 (90.95%)	48,129,177 (88.73%)	1,202,282 (2.22%)
QNF11-16a	49,792,618	47.99%	93.52%	45,230,328 (90.84%)	44,118,409 (88.60%)	1,111,919 (2.23%)
QNF11-16b	48,798,226	48.00%	93.40%	44,299,094 (90.78%)	43,175,218 (88.48%)	1,123,876 (2.30%)
QNF11-16c	44,926,024	47.93%	93.65%	882,404 (91.00%)	39,930,744 (88.88%)	951,660 (2.12%)

**Table 3:** Sequences of the primers used to amplify the fragment flanking SNP-5.

Primer Sequences		Products Length	Initial Position	Terminal Position
Forward (5′-3′)	Reverse (5′-3′)			
CTGATGTTGAGAAACGTCTAATGC	AGGGAGTAGTAAGTTTTGATGTTTC	266 bp	4,029,130 bp	4,029,395 bp

**Table 4:** Allelic variation of SNP-5 among cauliflower breeding lines detected in the present study.

Accessions	Material Type	Phenotype	Genotype of SNP-3	Genotype of SNP-5
QNF11-3	Inbred line	Riceyness	C/C	T/T
QNF11-16	Inbred line	Non-riceyness	G/G	C/C
ZA3005	DH line	Non-riceyness	C/C	C/C
ZA4279-1	Inbred line	Riceyness	G/G	T/T
ZA18601	Inbred line	Non-riceyness	G/G	C/C
ZA221-6	Inbred line	Non-riceyness	G/G	C/C
ZA4260	Inbred line	Riceyness	C/C	T/T
ZA4257	Inbred line	Non-riceyness	C/G	C/C
ZA4101	Inbred line	Non-riceyness	C/G	C/C
ZA3201-1	DH line	Non-riceyness	G/G	C/C
ZA3203-61	DH line	Riceyness	C/C	T/T
ZA4715-1	Inbred line	Non-riceyness	G/G	C/C

## Data Availability

All the sequence data generated in this research was deposited in the Sequence Read Archive database (www.ncbi.nlm.nih.gov/sra) at the NCBI (National Center for Biotechnology Information) under accession numbers: SRR9663134, SRR9663135, SRR9663136, SRR9663137, SRR9663138, SRR9663139 for BSA-seq and SRR9703244, SRR9703245, SRR9703246, SRR9703247 for RNA-seq.

## Supplementary Materials

Supplementary materials can be found at https://www.mdpi.com/1422-0067/21/6/1999/s1. Figure S1: Normal cauliflower curd (A) and typical ricey curd (B), Figure S2: A pedigree diagram of QNF11-33 and QNF11-16, Table S1: Detailed informations of the cadidate SNPs and corresponding genes, Table S2: All the differentially expressed genes and their expresion levels in six samples.
