# Supplementary material for: Identification of Candidate Genes Involved in Curd Riceyness in Cauliflower
Source: Int J Mol Sci. 2020 Mar 15;21(6):1999. doi: 10.3390/ijms21061999 (PMC7139996; doi:10.3390/ijms21061999)
Supplement: Supplementary file 1 [file ijms-21-01999-s001.zip › figure S1.pdf]

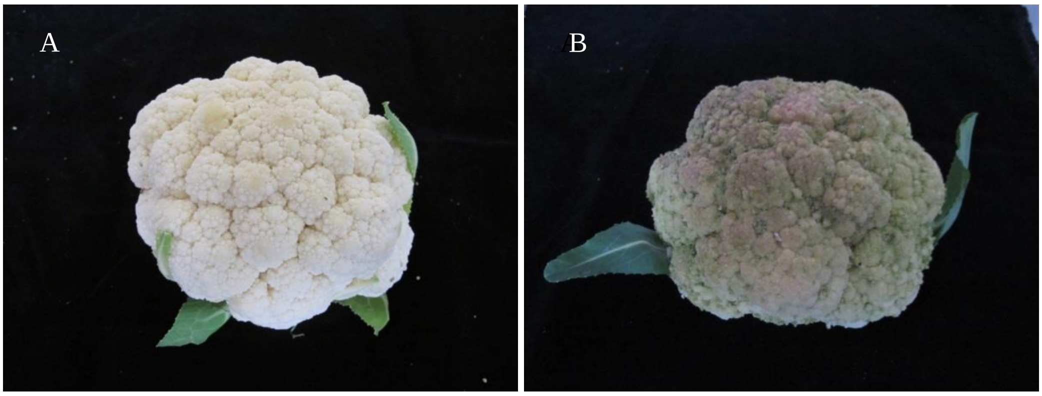

Fig. S1 Normal cauliflower curd (A) and typical ricey curd (B). The riceyness may appear milky white, green, purple or other colors under different temperature, light and other environmental conditions.
