# Supplementary figures and images for: Identification of Candidate Genes Involved in Curd Riceyness in Cauliflower
Source: Int J Mol Sci. 2020 Mar 15;21(6):1999. doi: 10.3390/ijms21061999 (PMC7139996; doi:10.3390/ijms21061999)

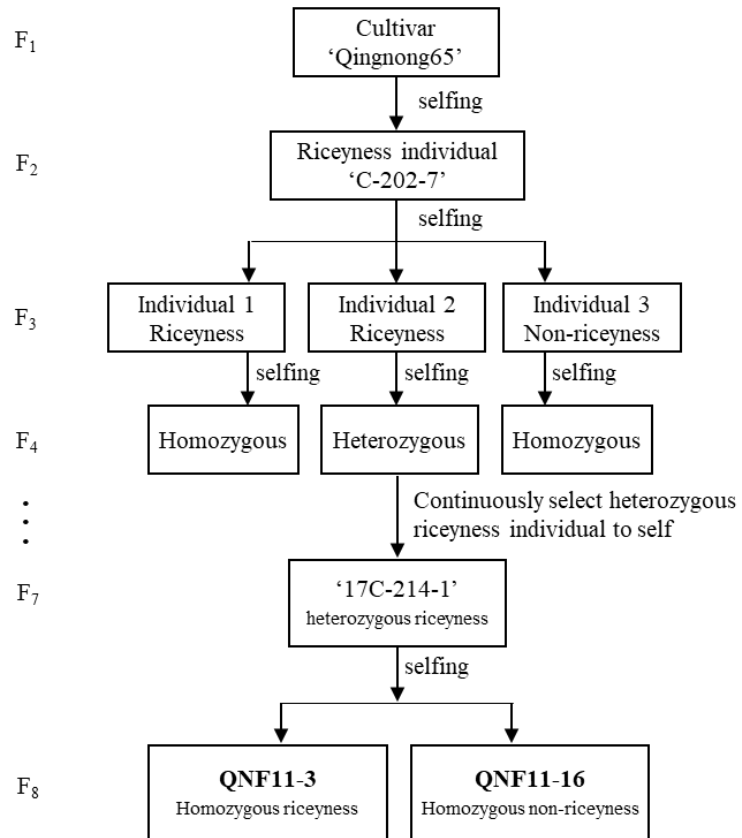

**Fig. S2 A** pedigree diagram of QNF11-3 and QNF11-16.

Supplement: Supplementary file 1 [file ijms-21-01999-s001.zip › figure S2.pdf]
